# Quality of Life Among End-Stage Renal Disease Patients Undergoing Maintenance Hemodialysis at King Abdulaziz Hospital, Kingdom of Saudi Arabia: A Cross-Sectional Study

**DOI:** 10.3390/healthcare14142071

**Published:** 2026-07-10

**Authors:** Nuha Eid Alotaibi, Anna Joyce Pitsoane, Felipe G. Martinez, Ramy I. Abulikailik

**Affiliations:** 1 Nursing Services, Hemodialysis, King Abdulaziz Hospital, Ministry of National Guard Health Affairs (MNGHA), Al Ahsa 36428, Saudi Arabia; pitsoanean@mngha.med.sa (A.J.P.); martinezfe@mngha.med.sa (F.G.M.J.); 2 King Abdullah International Medical Research Centre (KAIMRC), Al Ahsa 36428, Saudi Arabia; 3 Department of Nephrology, King Abdulaziz Hospital, Ministry of National Guard Health Affairs (MNGHA), Al Ahsa 36428, Saudi Arabia

**Keywords:** end-stage renal disease, hemodialysis, quality of life, WHOQOL-BREF, night shift dialysis, Saudi Arabia

## Abstract

**Highlights:**

**What are the main findings?**

- Patients undergoing maintenance hemodialysis experienced the greatest impairment in the physical and social domains of quality of life, whereas psychological and environmental domains showed comparatively higher scores.
- Female sex, lower educational level, and night-shift dialysis were associated with poorer physical quality-of-life outcomes.

**What are the implications of the main findings?**

- Routine assessment of quality of life should be incorporated into hemodialysis care to facilitate early identification of vulnerable patients and guide individualized management.
- Patient-centered dialysis scheduling together with psychosocial support and patient education may improve physical well-being and overall quality of life in patients with ESRD.

**Abstract:**

**Background/Objectives:** End-stage renal disease (ESRD) profoundly affects patients’ daily functioning and well-being, particularly among individuals receiving long-term hemodialysis. Evaluating quality of life (QoL) is therefore essential for understanding the broader impact of ESRD and its treatment. This study aimed to assess QoL and identify demographic and treatment-related factors associated with QoL among patients undergoing maintenance hemodialysis at a regional dialysis center in Al Ahsa, Eastern Saudi Arabia. **Methods:** A cross-sectional study was conducted among 79 adult patients receiving maintenance hemodialysis for at least three months at King Abdulaziz Hospital, Al Ahsa. Quality of life was assessed using the WHOQOL-BREF questionnaire, with domain scores transformed to a 0–100 scale. Statistical analyses included descriptive statistics, independent *t*-tests, one-way ANOVA, Spearman’s correlation analysis, and multinomial logistic regression. **Results:** The physical domain demonstrated the lowest QoL score (49.69 ± 18.77), followed by the social domain (48.02 ± 24.92), whereas the psychological (63.83 ± 19.15) and environmental (67.56 ± 19.27) domains showed comparatively higher scores. Female sex, lower educational attainment, and night-shift dialysis were associated with poorer physical QoL. Significant positive correlations were observed among all QoL domains (*p* < 0.01), indicating strong interrelationships between different aspects of quality of life. **Conclusions:** Patients undergoing maintenance hemodialysis experienced substantial impairment in the physical and social dimensions of quality of life, while psychological and environmental well-being remained relatively preserved. These findings support the routine assessment of QoL in dialysis care and highlight the need for targeted interventions, including optimized dialysis scheduling, structured physical activity programs, and psychosocial support, to improve overall patient well-being with ESRD.

## 1. Lay Summary

People with end-stage kidney disease often depend on regular hemodialysis to stay alive. While dialysis is lifesaving, it also affects daily life in many ways, including in terms of energy levels, sleep, mood, and social activities. This study investigated how patients receiving maintenance hemodialysis in Al Ahsa, Saudi Arabia, experience their quality of life across the physical, psychological, social, and environmental domains.

Overall, patients reported experiencing the greatest difficulties in the physical and social areas of their lives. Many patients experienced fatigue, reduced stamina, and limitations pertaining to everyday activities. Social participation was also affected, especially among women and older adults. Despite these challenges, the psychological and environmental well-being were improved, likely reflecting strong family support and reliable access to dialysis services.

One of the most important findings of this study pertains to the impact of dialysis timing, particularly with respect to night shift sessions. Unlike in many Western countries, in which dialysis is usually scheduled during daytime hours, night dialysis is typically used in busy centers to accommodate patient demand. However, attending dialysis late at night can disturb patients’ normal sleep patterns, lead to persistent daytime tiredness, and make it more difficult for them to maintain regular family and social routines. Traveling to and from the hospital at night may also be more difficult and stressful for both patients and their caregivers. Over time, these factors can reduce patients’ physical strength, motivation, and overall satisfaction with their treatment schedules.

These findings highlight the fact that the timing of dialysis is not merely an organizational issue but also a patient-centered concern. Adjusting dialysis schedules when possible, improving nighttime support services, and considering patients’ preferences may help reduce the burden associated with nocturnal sessions. By recognizing how night shift dialysis affects patients’ daily lives, health care providers can design more supportive and flexible care plans that can improve both patients’ well-being and their long-term treatment adherence.

In simple terms, this study reveals that dialysis keeps patients alive; however, the manner in which such treatment is scheduled—especially during the night—can strongly influence how well patients live. Efforts to understand and address these challenges are essential for improving the overall quality of life of people undergoing long-term hemodialysis.

## 2. Introduction

Chronic kidney disease (CKD) is a major global public health challenge affecting approximately one in ten adults worldwide. It is associated with substantial morbidity, premature mortality, and healthcare expenditure. The increasing prevalence of diabetes mellitus, hypertension, obesity, and population aging has contributed to a steady rise in the number of individuals progressing to end-stage renal disease (ESRD), creating expanding demand for kidney replacement therapy. Despite advances in renal transplantation and home-based dialysis modalities, maintenance hemodialysis remains the most frequently used treatment for patients with ESRD worldwide [1,2,3].

Although maintenance hemodialysis effectively replaces essential renal function and prolongs survival, it does not restore normal health. Patients receiving long-term hemodialysis are exposed to a complex combination of disease-related and treatment-related burdens, including persistent fatigue, sleep disturbances, pruritus, pain, muscle cramps, dietary and fluid restrictions, vascular access complications, prolonged post-dialysis recovery, and frequent hospitalizations. These challenges often interfere with patients’ ability to maintain employment, fulfill family responsibilities, participate in social activities, and preserve their independence, resulting in significant impairment of overall well-being and daily functioning [4,5,6]. Recent evidence has also demonstrated that impaired health-related quality of life (HRQoL) is associated with increased hospitalization and mortality among patients undergoing maintenance hemodialysis, emphasizing that successful dialysis care should be evaluated using patient-reported outcomes in addition to conventional biochemical and clinical indicators [4,5,6].

The World Health Organization (WHO) defines quality of life (QoL) as an individual’s perception of their position in life within the context of the culture and value systems in which they live, and in relation to their goals, expectations, standards, and concerns [7,8,9]. This definition recognizes QoL as a multidimensional construct encompassing physical health, psychological well-being, social relationships, level of independence, environmental circumstances, and personal beliefs. Consequently, QoL has become a fundamental patient-centered outcome in nephrology because it reflects the overall impact of chronic kidney disease and dialysis treatment from the patient’s perspective rather than focusing solely on laboratory parameters or dialysis adequacy measures [7,8,9].

Growing evidence indicates that QoL among patients receiving maintenance hemodialysis is influenced by a complex interaction of demographic, socioeconomic, clinical, and treatment-related factors. Advanced age, female sex, lower educational attainment, unemployment, anemia, malnutrition, prolonged dialysis vintage, poor sleep quality, multiple comorbidities, vascular access complications, depression, and dialysis-related symptoms have all been associated with poorer physical and psychological well-being [4,5,6,10,11,12,13,14,15,16,17]. Several of these determinants are potentially modifiable, highlighting the importance of routinely assessing QoL to identify vulnerable patients and support individualized interventions aimed at improving both clinical outcomes and patient satisfaction [4,5,6,13,14,15,16,17,18,19,20].

Although kidney disease-specific instruments such as the KDQOL-36 provide a detailed assessment of dialysis-related symptoms, treatment burden, and kidney disease-specific concerns, the WHOQOL-BREF was selected for the present study because the primary objective was to evaluate overall QoL across broad physical, psychological, social, and environmental domains rather than focusing exclusively on kidney disease-specific symptoms [8,9,16,21].

The WHOQOL-BREF has been internationally validated, is brief, is practical for clinical research, and allows comparison across different populations, chronic diseases, and cultural settings. This broader perspective is particularly relevant in hemodialysis patients, whose QoL is shaped not only by dialysis-related symptoms but also by family roles, social support, emotional well-being, environmental conditions, employment, and access to healthcare services [8,9,16,17,21].

Recent studies using WHOQOL-BREF and other validated QoL instruments have confirmed that patients receiving maintenance hemodialysis often report reduced scores across multiple domains, particularly physical health and social functioning. Physical limitations are commonly related to fatigue, reduced mobility, pain, sleep disturbance, dialysis recovery time, and comorbidity burden, whereas psychological and social domains may be affected by anxiety, depressive symptoms, dependency on treatment, reduced social interaction, and perceived loss of autonomy. These findings support the need for multidimensional QoL assessment rather than relying on a single global measure of health status [10,11,12,13,14,15,16,17,20].

Dialysis shift timing is another clinically relevant factor that may influence QoL. Patients receiving hemodialysis at different times of the day may experience differences in sleep quality, fatigue, recovery time, social participation, family responsibilities, work schedule, and overall daily functioning [22,23,24,25,26]. For example, early morning sessions may interfere with sleep and pre-dialysis preparation, afternoon sessions may disrupt family or occupational activities, and late evening or night shifts may affect sleep–wake rhythm and post-dialysis recovery. These patterns suggest that dialysis scheduling may influence patient experience beyond dialysis adequacy or biochemical control alone [22,23,24,25,26]. Recent evidence has increasingly explored the relationship between dialysis shift, sleep quality, recovery time, and QoL. Studies have reported that dialysis shift timing may be associated with sleep problems, fatigue, and differences in patient-reported outcomes among hemodialysis patients [22,23]. Prolonged dialysis recovery time has also been associated with poorer QoL [24], suggesting that the period after dialysis is an important but often under-recognized component of the patient experience. In addition, studies of in-center nocturnal hemodialysis have shown improvements in selected HRQoL domains, further supporting the concept that dialysis timing and treatment structure may meaningfully affect patient-centered outcomes [25,26]. Therefore, evaluating QoL across dialysis shifts may provide practical information for dialysis units, particularly when planning schedules, counseling patients, and identifying groups who may require additional support. However, dialysis shift findings should be interpreted carefully because they may be influenced by several clinical factors, including dialysis vintage, comorbidity burden, hemoglobin level, serum albumin level, sleep quality, vascular access type, and intradialytic symptoms. These factors may act as confounders and should be considered in future studies with more comprehensive clinical datasets.

In Saudi Arabia, the burden of ESRD has increased considerably over the past two decades, primarily because of the high prevalence of diabetes mellitus, hypertension, obesity, and an aging population [13,14,15]. Consequently, the number of patients requiring maintenance hemodialysis continues to rise, placing substantial demands on healthcare resources while emphasizing the need to optimize patient-centered outcomes in addition to clinical survival. Previous studies conducted in Saudi Arabia have consistently demonstrated impaired QoL among patients undergoing maintenance hemodialysis, with physical health generally being the most affected domain. Factors including female sex, unemployment, lower educational attainment, older age, and the presence of multiple comorbidities have been associated with poorer QoL outcomes [12,13,14,15,19]. However, most available studies have been conducted in large metropolitan dialysis centers, and evidence from regional healthcare settings remains relatively limited.

Al-Ahsa is one of the major regions of Saudi Arabia and serves a diverse population with varying socioeconomic characteristics and healthcare needs. Despite the growing number of patients receiving maintenance hemodialysis in this region, comprehensive evaluation of QoL using internationally validated instruments remains limited. Furthermore, few studies have examined whether dialysis shift schedules influence QoL within routine clinical practice. Since dialysis scheduling affects patients’ daily activities, employment, sleep patterns, family responsibilities, and social participation, understanding its relationship with QoL may provide valuable information for improving patient-centered dialysis services and optimizing resource allocation within dialysis units [22,23,24,25,26].

Although numerous studies have evaluated QoL among hemodialysis patients worldwide, important knowledge gaps remain. In particular, limited evidence is available regarding the combined influence of dialysis shift schedules and sociodemographic characteristics on the different domains of QoL among patients receiving maintenance hemodialysis in Saudi Arabia. Moreover, comparisons between physical, psychological, social, and environmental domains using the WHOQOL-BREF instrument have been infrequently reported in regional dialysis populations. Addressing these gaps is important because identifying factors associated with impaired QoL may facilitate targeted interventions, individualized supportive care, and quality-improvement initiatives aimed at enhancing patient well-being and long-term outcomes [10,11,12,13,14,15,16,17].

Therefore, the present study aimed to evaluate the quality of life of adult patients with ESRD undergoing maintenance hemodialysis at King Abdulaziz Hospital, Al-Ahsa, Saudi Arabia, using the World Health Organization Quality of Life-BREF (WHOQOL-BREF) instrument. In addition, the study sought to examine the associations between QoL domains and patients’ sociodemographic characteristics, as well as dialysis shift schedules, to identify factors associated with poorer QoL and generate evidence that may inform patient-centered clinical practice, service planning, and future research in the Saudi hemodialysis population.

### 2.1. Aim of This Study

This study aimed to evaluate the quality of life among patients with end-stage renal disease receiving maintenance hemodialysis using the WHOQOL-BREF instrument. It also sought to assess the association between sociodemographic, clinical, and treatment-related characteristics and quality of life outcomes.

### 2.2. Study Hypotheses

This study was based on the following hypotheses:

- Patients receiving maintenance hemodialysis experience impairments in quality of life across the physical, psychological, social, and environmental domains of the WHOQOL-BREF;
- Quality of life scores differ according to sociodemographic characteristics, including age, sex, and educational level;
- Clinical and treatment-related factors, including dialysis shift, are significantly associated with quality of life outcomes among hemodialysis patients.

## 3. Materials and Methods

### 3.1. Study Design and Setting

While several studies have evaluated quality of life among hemodialysis patients in Saudi Arabia, data from the Al Ahsa region remain limited. Al Ahsa represents a unique population within the Eastern Province, with distinct demographic, cultural, and healthcare characteristics that may influence patients’ quality of life. Therefore, it is important to assess quality of life among hemodialysis patients in this regional setting in order to provide locally relevant evidence and guide patient-centered care.

This study was conducted at the Dialysis Unit of King Abdulaziz Hospital, Al Ahsa, Kingdom of Saudi Arabia, which operates under the auspices of the National Guard Health Affairs. King Abdulaziz Hospital is a tertiary, 325-bed teaching hospital. The dialysis unit served approximately 100 patients and performed approximately 15,362 hemodialysis sessions in 2024.

In our dialysis unit, services were divided into four shifts—morning, afternoon, evening, and night—to ensure that all patients receive their dialysis treatments according to schedule. Patients generally tolerated the morning, afternoon, and evening shifts well, but the night shift was associated with unique challenges.

This shift typically began at 12:30 a.m. and ended at approximately 5:00 a.m., thus making it difficult not only for patients—who must adjust their sleep patterns—but also for their families, who often accompany them or provide transportation assistance during these late hours. However, some patients reported that they appreciated the night shift because of the quieter environment and increased privacy that it offered. Despite these challenges, night shifts remain essential to the goals of accommodating a large number of patients who require regular hemodialysis and maintaining continuity of care within the unit.

### 3.2. Sample Size Calculation

The required sample size was calculated using the Raosoft^®^ Sample Size Calculator (Raosoft, Inc., Seattle, WA, USA) https://www.raosoft.com/samplesize.html accessed on 1 August 2025. Based on a total hemodialysis population of 96 patients in the dialysis unit, a confidence level of 95%, a margin of error of 5%, and a response distribution of 50%, the minimum required sample size was estimated to be 74 participants.

### 3.3. Sampling Strategy

**Figure 1:**
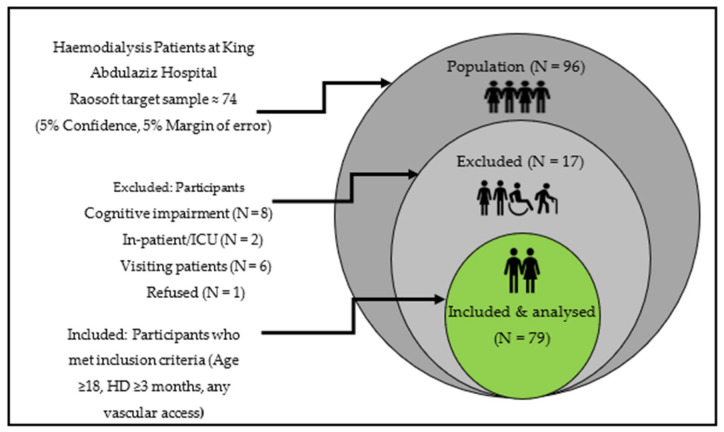
Sampling structure indicating the total dialysis population, excluded participants, and the final sample included in the analysis.

All patients who were receiving maintenance hemodialysis at King Abdulaziz Hospital during the study period were screened for eligibility. A consecutive sampling approach was employed, whereby all patients meeting the predefined inclusion criteria and none of the exclusion criteria were invited to participate. Of the total dialysis population of 96 patients, 17 were excluded according to predefined criteria or declined participation, resulting in a final analytical sample of 79 participants (see Figure 1).

Our study was a single-center, cross-sectional study that focused on 79 patients who were receiving maintenance hemodialysis three times per week (including 46 females and 33 males). The inclusion criteria focused on adults aged 18 years and older, regardless of their vascular access type, who had been receiving chronic hemodialysis for at least three months.

The exclusion criteria included patients with severe cognitive impairments (e.g., dementia, intellectual disability, or comatose state), patients with critical illnesses that required inpatient or intensive care services, patients who refused to participate in this research, and transient or visiting dialysis patients from other institutions (visiting dialysis patients were defined as individuals who were temporarily dialyzed at King Abdulaziz Hospital during travel, referral, or short-term transfer from another dialysis center). These patients were excluded to ensure a homogeneous study population consisting of patients routinely receiving maintenance hemodialysis at the study center. A total of 17 patients were excluded: 8 as a result of severe cognitive impairment, 2 as a result of critical illness, 6 in light of their identity as transient dialysis patients, and 1 for declining to participate in this research (see Figure 1).

Data collection took place between August 2025 and September 2025 on the basis of face-to-face structured interviews that were conducted by the researchers during the patients’ regular dialysis sessions. Because many patients were elderly or had limited literacy, the interviews were conducted in Arabic and lasted an average of 30 min. Patients were interviewed according to their dialysis schedules (i.e., during the morning, afternoon, evening, and night shifts) to minimize disruptions to the clinical workflow.

Before participation, written informed consent was obtained from all participants, who were assured that their responses would remain anonymous and confidential. The purpose of the study and the content of the questionnaire (WHOQOL-BREF instrument) were explained in simple and clear language to improve patients’ understanding and ensure accurate responses.

### 3.4. Ethical Considerations

Ethical approval for this study was obtained from the Institutional Review Board (IRB) of King Abdullah International Medical Research Centre (KAIMRC) (Approval No: (IRB No: 00000197525) on 4 August 2025, before the study was conducted. The study was conducted in accordance with the principles of the Declaration of Helsinki and all relevant ethical guidelines for research involving human participants. All participants provided written informed consent prior to enrolment in this research, and confidentiality was strictly maintained throughout the research process.

### 3.5. Tools and Procedures Used to Assess Quality of Life

The World Health Organization Quality of Life-BREF (WHOQOL-BREF) was the main tool used to assess the quality of life of end-stage renal disease patients undergoing maintenance hemodialysis at King Abdulaziz Hospital, Ahsa, Kingdom of Saudi Arabia.

This is a standardized method for assessing quality of life; it was developed by the World Health Organization as a concise version of the original WHOQOL-100 and offers a reliable and cross-culturally valid assessment of an individual’s perceived quality of life across diverse cultural and clinical contexts. The WHOQOL-BREF contains 26 items, including 2 general items used to evaluate overall quality of life and satisfaction with health, as well as 24 items distributed across four domains: physical health, psychological well-being, social relationships, and environmental health. Each item is scored on a five-point Likert scale, and the domain scores are computed by summing the items within each domain during the WHOQOL-BREF scoring process. It is important to clarify how the domain scores are generated and interpreted. Each domain is first calculated by summing the responses to the associated items to obtain a raw score. Because the raw totals differ in range from one domain to another, the WHO recommends that these values should be converted in line with a standardized 0–100 scale. This transformation is performed by adjusting the raw totals to the WHOQOL-BREF’s 4–20 metric and subsequently applying a linear conversion formula to ensure that all the domains share the same 0–100 range. Presenting the results in this format facilitates easier comparisons, both across different domains and with findings from other studies. On this scale, higher values indicate better perceived quality of life, whereas lower values indicate greater limitations or dissatisfaction within the domain in question. The inclusion of a brief explanation of this scoring process (possibly as an appendix) helps ensure transparency and allows readers to interpret the results more accurately.

The WHOQOL-BREF questionnaire was administered in Arabic using a previously translated and validated Arabic version. This version has demonstrated satisfactory psychometric properties and has been used in Arabic-speaking populations. Therefore, no additional translation was required for the present study.

The multidimensional structure, brevity, and proven psychometric robustness of the WHOQOL-BREF make it a suitable instrument for efforts to assess the quality of life of patients with end-stage renal disease receiving maintenance hemodialysis.

### 3.6. Statistical Analysis

All statistical analyses were conducted using IMB SPSS Statistics software for Windows, version 20.0 (IBM Corp., Armonk, NY, USA). Both descriptive and inferential statistical approaches were employed. First, the data were examined to ensure their completeness and consistency, and any missing or outlier values were evaluated. The distribution of continuous scores was assessed by performing the Shapiro–Wilk test to determine whether parametric assumptions were met. Because the WHOQOL-BREF domain scores are based on ordinal questionnaire responses and tend to deviate from a normal distribution in clinical populations, the transformed scores were treated as continuous in the comparison of mean differences across groups.

Data were reviewed for completeness before analysis. Participants with incomplete questionnaires were not included in the final dataset. The final analytical sample consisted of 79 participants with complete data; therefore, no imputation procedures were required for missing values.

Descriptive statistics, including sex, age group, level of education, employment status, marital status, and dialysis shift schedule, were used to summarize the participants’ sociodemographic profiles. Frequencies and percentages were used for categorical variables, whereas means and standard deviations (SDs) were used to summarize continuous QoL domain scores.

To examine the relationships between sociodemographic factors and QoL outcomes, a one-way analysis of variance (ANOVA) was performed to compare mean QoL domain scores among variables pertaining to three or more categories, such as age group, level of education, and dialysis shift. For variables that include two categories, such as sex, independent sample t tests were performed. Where the assumptions for chi-square testing were not met because of small expected cell counts, Fisher’s exact test was used to evaluate the associations between categorical variables and categorized QoL levels, thus facilitating more precise *p* value estimations.

Prior to multinomial logistic regression analysis, WHOQOL-BREF domain scores were categorized into predefined quality of life levels (poor, moderate, good, and very good). These categorized domain scores were then used as the dependent variables in the regression models.

Multinomial logistic regression analysis was performed to identify independent predictors of quality of life (QoL) across each WHOQOL-BREF domain. For each domain, QoL scores were categorized into ordinal categories, with the “very good” category designated as the reference outcome. Variables demonstrating a significance level of *p* < 0.25 in the bivariate analysis, together with clinically relevant variables identified from the literature, were entered into the multivariable model. The same set of sociodemographic and work-related characteristics was included across all models to ensure consistency. Adjusted odds ratios (AORs) with 95% confidence intervals 95% CIs and corresponding *p*-values were reported. A two-sided *p* value < 0.05 was considered statistically significant.

In addition, the Spearman rank correlation coefficient (ρ) was used to assess the strength and direction of the relationships among the four QoL domains in light of their ordinal nature and potential interdependence. Across all the statistical tests, a *p* value < 0.05 was considered to indicate statistical significance.

## 4. Results

### 4.1. Demographic Characteristics

A total of 79 patients who were receiving maintenance hemodialysis were included, 58.2% of whom were female, and 41.8% were male. Most participants were in the age range of 50–70 years (51.9%), followed by >70 years (25.3%), 30–50 years (20.3%), and 20–30 years (2.5%). Nearly half of the cohort was illiterate (44.3%), whereas 11.4% had a university-level education. The majority of participants were unemployed (62.0%) or retired (32.9%). Most patients were married (63.3%). Dialysis sessions were relatively evenly distributed across the morning (31.6%), afternoon (22.8%), evening (22.8%), and night (22.8%) shifts (Table 1).

### 4.2. Reliability Assessment of the WHOQOL-BREF Instrument

The internal reliability and stability of the WHOQOL-BREF instrument in this sample were evaluated using Cronbach’s alpha coefficient, which documented high internal consistency (α = 0.946), thus indicating that the questionnaire items reliably measured the intended QoL constructs. Raw domain scores were calculated in accordance with the guidelines stipulated by the WHO, converted to mean scores, and subsequently transformed to the 0–100 scale, in which higher values indicate a more favorable quality of life. For the sake of interpretive clarity, these scores were further divided into very poor (<50), poor, moderate (50–74), good (≥75), and very good, thus providing a structured representation of QoL levels across participants.

### 4.3. Distribution of QoL Ratings Across Domains

**Figure 2:**
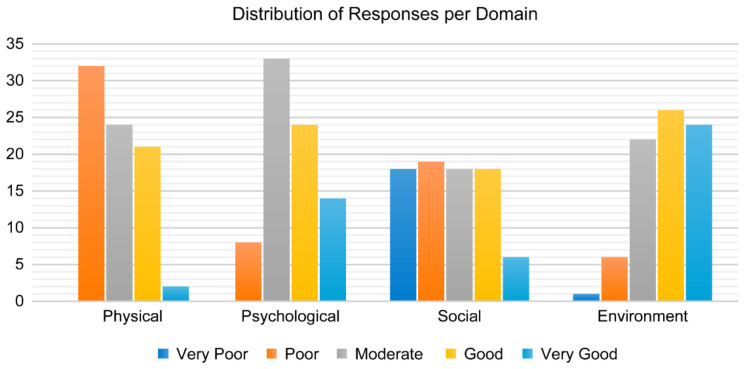
Distribution of participants’ quality of life ratings across the four WHOQOL-BREF domains.

Quality of life ratings varied across the different WHOQOL-BREF domains. The physical domain was associated with the poorest outcomes, with 40.5% of participants reporting poor QoL and only 2.5% reporting very good QoL. The psychological domain received better scores, with most participants reporting moderate (41.8%) or good (30.4%) QoL. The social domain scores were more evenly distributed, whereas the environmental domain scores were the most favorable, with 63.2% of participants reporting good or very good QoL in this regard (Table 2) (see Figure 2).

### 4.4. Domain Mean and Transformed Scores

**Figure 3:**
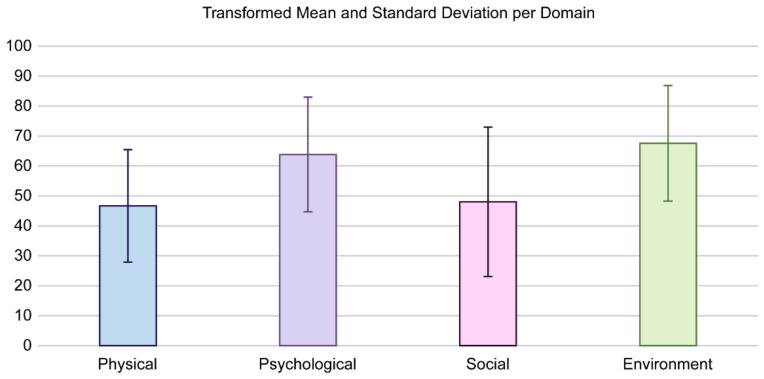
Mean and transformed WHOQOL-BREF domain scores among hemodialysis patients.

Transformed mean scores ranged from 48.02 to 67.56. The physical domain exhibited the lowest score (49.69 ± 18.77), followed by the social domain (48.02 ± 24.92). Higher scores were observed for the psychological (63.83 ± 19.15) and environmental (67.56 ± 19.27) domains, thus indicating better psychological well-being and environmental satisfaction among the participants in these contexts (Table 3) (see Figure 3).

### 4.5. Correlations Among the QoL Domains

Spearman’s correlation analysis revealed strong positive correlations among all the QoL domains (*p* < 0.001). The psychological domain exhibited the strongest correlations with the environmental (r = 0.774), physical (r = 0.729), and social (r = 0.723) domains, thus highlighting the interdependence of quality of life components in hemodialysis patients (Table 4).

### 4.6. Associations Between Sociodemographic Characteristics and QoL Domains

The associations between sociodemographic variables and WHOQOL-BREF domains were examined by performing a one-way ANOVA. Domain scores were transformed in line with a 0–100 scale, in which higher scores indicated better quality of life. As a result of the limited subgroup sizes in some domains, statistical testing was not always feasible.

#### 4.6.1. Social QoL Domain

**Figure 4:**
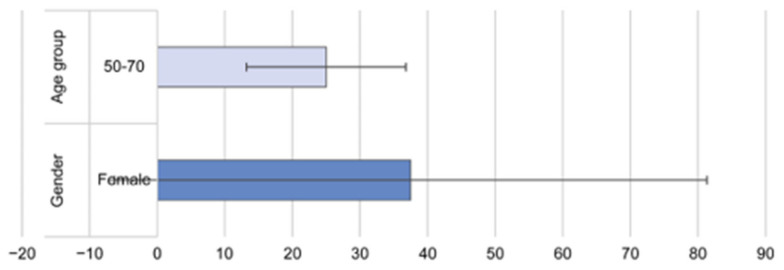
Associations between sociodemographic characteristics and the social QoL domain.

Data availability for the social domain was limited, thus restricting meaningful comparisons across most sociodemographic variables. Sex exhibited a significant association, with females reporting lower social QoL scores than males (*p* = 0.04). No statistically significant differences were observed with respect to age, education, employment status, marital status, or dialysis shift (*p* > 0.05). The associations between sociodemographic characteristics and the social QoL domain are presented in Table 5 (see Figure 4).

#### 4.6.2. Psychological QoL Domain

**Figure 5:**
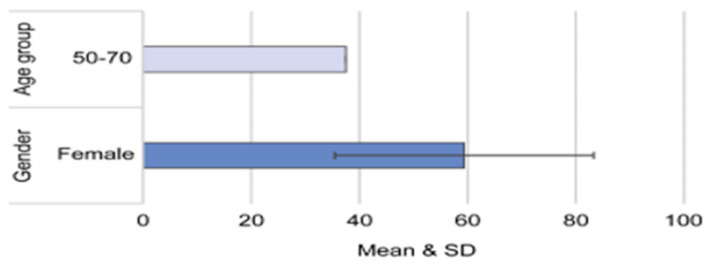
Associations between sociodemographic characteristics and the psychological QoL domain.

Psychological QoL data were also limited across subgroups. Age and level of education were significantly associated with psychological QoL (*p* = 0.038 for both), whereas sex, employment status, marital status, and dialysis shift were not significantly related to that factor (*p* > 0.05). Detailed results regarding the associations between sociodemographic characteristics and the psychological QoL domain are presented in Table 6 (see Figure 5).

#### 4.6.3. Physical QoL Domain

**Figure 6:**
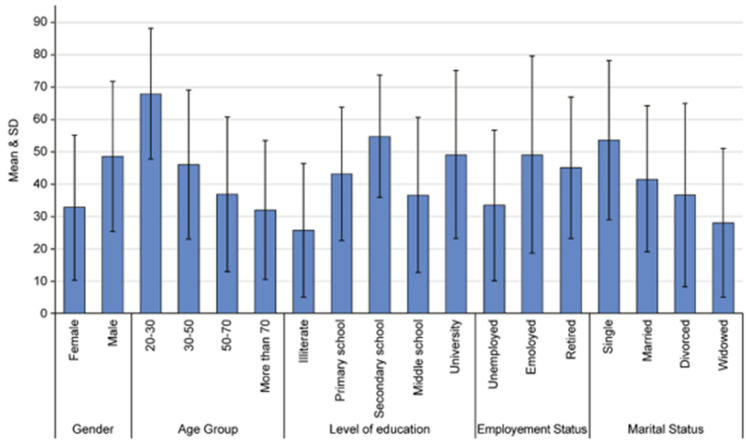
Associations between sociodemographic characteristics and the physical QoL domain.

The physical domain yielded sufficient data to conduct a full analysis. In comparison with female patients, male patients obtained significantly higher physical QoL scores (*p* = 0.005). Level of education exhibited a strong gradient, ranging from lower scores among illiterate participants to higher scores among individuals with a secondary or university education (*p* < 0.001). Dialysis shift was also significantly associated with physical QoL (*p* = 0.025), as lower scores were reported among night shift patients. Age, employment status, and marital status did not reach the level of statistical significance (*p* > 0.05). The associations between sociodemographic variables and the physical QoL domain are summarized in Table 7 (see Figure 6).

#### 4.6.4. Environmental QoL Domain

**Figure 7:**
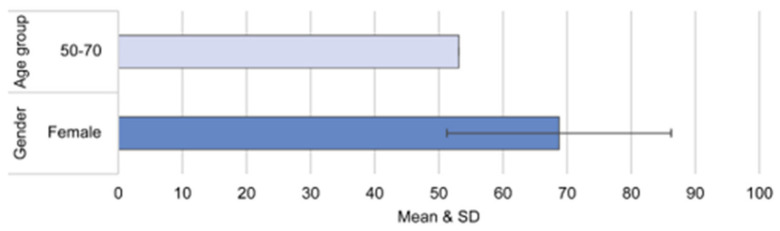
Associations between sociodemographic characteristics and the environment QoL domain.

Environmental QoL data were limited to a few subgroups, thus preventing robust statistical comparisons. Descriptively, environmental QoL scores were higher than those observed in the social and physical domains, thus suggesting relatively favorable perceptions of health care access, living conditions, and financial resources among respondents (Table 8) (see Figure 7).

##### Summary of Domain Findings

Among the four domains, the physical domain was the only one with enough data for full statistical analysis. Significant differences were noted for sex and level of education. In the social, psychological, and environmental domains, the very small number of participants in each subgroup restricted comparative analysis.

Across all the domains, the general patterns were consistent: women, older patients, and participants with lower levels of education tended to report lower quality of life, particularly in the physical and social domains. These trends align with patterns reported internationally in hemodialysis populations.

#### 4.6.5. Multinomial Logistic Regression Results

Multinomial logistic regression was performed for each WHOQOL-BREF domain, with the QoL category “very good” set as the reference outcome. For each model, we included the same set of sociodemographic and work-related predictors that we used in the bivariate analysis, and we report adjusted odds ratios (ORs) with 95% confidence intervals (CIs).

##### Physical Domain

The multinomial logistic regression model for the physical QoL domain yielded a pseudo-R^2^ of 0.569. Education level was significantly associated with physical QoL categories in several comparisons. Compared with participants with university-level education, those with primary education had significantly lower odds of being classified in the very good physical QoL category in both the poor versus very good comparison (OR = 4.45 × 10^−8^, 95% CI: 7.80 × 10^−10^–2.54 × 10^−6^; *p* < 0.001) and the good versus very good comparison (OR = 2.39 × 10^−8^, 95% CI: 4.86 × 10^−10^–1.18 × 10^−6^; *p* < 0.001). Similar statistically significant associations were observed among participants with secondary-level education across multiple comparisons. No statistically significant associations were observed between dialysis shift and physical QoL categories after adjustment, as all comparisons involving afternoon, evening, and morning shifts yielded non-significant *p*-values. The complete multinomial logistic regression results for the physical QoL domain are presented in Table 9.

##### Psychological Domain

The multinomial logistic regression model for the psychological QoL domain yielded a pseudo-R^2^ of 0.334. Most sociodemographic variables were not significantly associated with psychological QoL categories, and the majority of age and education categories showed non-significant associations. Compared with participants with university-level education, illiterate participants had significantly higher odds of being classified in the moderate rather than the very good psychological QoL category (OR = 21.08; 95% CI: 1.98–224.02; *p* = 0.011). No statistically significant associations were observed for age group or dialysis shift after adjustment. The complete multinomial logistic regression results for the psychological QoL domain are presented in Table 10.

##### Social Domain

For the social QoL domain, the multinomial logistic regression model yielded a pseudo-R^2^ of 0.324. Female participants had significantly higher odds of being classified in the good rather than the very good social QoL category compared with male participants (OR = 38.72; 95% CI: 1.84–816.98; *p* = 0.019). No statistically significant associations were observed between employment status and social QoL categories after adjustment, and all comparisons involving employment status yielded non-significant results. The complete multinomial logistic regression results for the social QoL domain are presented in Table 11.

##### Environment Domain

The multinomial logistic regression model for the environmental QoL domain yielded a pseudo-R^2^ of 0.618. Compared with participants older than 70 years, those aged 50–70 years had significantly lower odds of being classified in the good rather than the very good environmental QoL category (OR = 0.128; 95% CI: 0.022–0.744; *p* = 0.022). Participants working the afternoon shift also had significantly lower odds of being classified in the good rather than the very good environmental QoL category compared with those working the night shift (OR = 0.062; 95% CI: 0.006–0.671; *p* = 0.022). No statistically significant associations were observed for the remaining education categories after adjustment. The complete multinomial logistic regression results for the environmental QoL domain are presented in Table 12.

### 4.7. Rationale for Selecting Studies for International Comparison

The selection of the three international studies included in this research was not arbitrary; rather, it was guided by methodological alignment and the need to ensure regional diversity. All the included papers used the WHOQOL-BREF tool, focused specifically on chronic hemodialysis patients, and reported domain-level scores in a manner consistent with our analytical structure. These studies were intentionally drawn from three different continents to illustrate variations in health care resources, cultural expectations, and dialysis delivery models; however, they should not be interpreted as nationally representative of their respective countries. Rather, they were selected solely because they met the predefined inclusion criteria, especially in the absence of recent national studies that have used the same instrument. This approach allowed us to situate our findings within a broader global context while simultaneously ensuring that the comparisons made in this research were methodologically sound and clinically meaningful.

**Figure 8:**
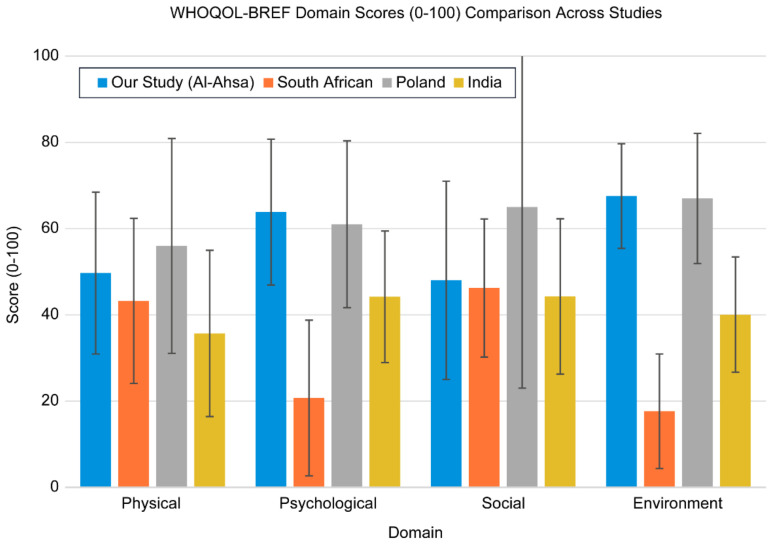
Comparison of WHOQOL-BREF domain scores across studies conducted in Saudi Arabia, Poland, India and South Africa. The values represent transformed scores (0–100 scale).

To conduct a situational analysis, we compared our results with those of comparable single-center international studies conducted in Poland, India, and South Africa to generate a global perspective regarding the quality of life of hemodialysis patients across different health care frameworks and socioeconomic backgrounds (see Figure 8).

## 5. Discussion

The present study presents a comprehensive evaluation of the quality of life (QoL) among patients with end-stage renal disease (ESRD) who were receiving maintenance hemodialysis at King Abdulaziz Hospital, Al Ahsa. We used the WHOQOL-BREF instrument to reveal that the QoL of this population is notably impaired, particularly in the physical and social domains, whereas their scores in the psychological and environmental domains are relatively high. These findings are consistent with recent international studies describing the multidimensional burden of ESRD on patients’ physical, psychological, and social functioning [11,16,17].

### 5.1. Overall QoL Patterns in the Al Ahsa Cohort

The pattern observed in this study—where the lowest scores were observed in the physical and social domains, whereas the highest scores were observed in the psychological and environmental domains—mirrors findings reported in other hemodialysis populations [17,18,27]. Physical QoL was significantly compromised in this cohort, as nearly half of the patients reported poor functioning. This finding is consistent with the well-documented symptom burden associated with maintenance hemodialysis, including fatigue, musculoskeletal pain, sleep disturbances, dietary restrictions, and reduced physical functioning [17,22,24].

The psychological QoL observed in our cohort was relatively well preserved. These higher psychological scores may be influenced by strong family support, cultural coping strategies, and spiritual resilience, all of which have been reported to facilitate emotional adaptation among patients receiving hemodialysis in Middle Eastern settings [15,19]. Family support may also extend beyond emotional resilience, as caregiver well-being has been shown to influence the overall care experience and adaptation of patients receiving maintenance hemodialysis, highlighting the interdependence between patient and caregiver outcomes [28].

Social QoL was notably lower, particularly among women, who accounted for the majority of “Very Poor” ratings. These findings may reflect disruption of traditional family and social roles together with reduced social participation experienced by patients receiving long-term hemodialysis, as reported in recent regional studies [13,14,15,19].

Environmental QoL was the strongest domain in our study, suggesting that patients benefited from accessible dialysis services, transportation, and financial support. Similar findings have been reported in European hemodialysis populations receiving care within well-developed healthcare systems [29].

These overall domain-level patterns were further clarified by multivariable analyses, which demonstrated that educational level, sex, age group, and dialysis shift independently influenced domain-specific QoL outcomes within this cohort.

### 5.2. Sociodemographic Determinants

Education was identified as the strongest determinant of QoL, which is consistent with studies demonstrating that greater health literacy is associated with improved treatment adherence, self-management, coping ability, and overall quality of life among patients receiving maintenance hemodialysis [13,14,19]. Age was significantly associated with the psychological and environmental domains, with older adults experiencing poorer outcomes. Sex was strongly associated with social QoL, supporting previous reports that female patients with ESRD experience greater social vulnerability and treatment-related psychosocial burden, particularly in conservative cultural settings [15,19].

While these findings describe the overall distribution of quality of life domains within the Al Ahsa cohort and their alignment with international patterns, multivariable analyses provided further insights into the underlying determinants of these outcomes. Specifically, level of education, sex, age group, and dialysis shift independently influenced domain-specific QoL scores, thus highlighting the fact that the observed patterns are shaped by both contextual and individual-level factors.

Although educational attainment emerged as an important determinant of domain-specific quality of life, several educational subgroups included relatively few participants, resulting in unstable odds ratio estimates. Consequently, these associations should be interpreted cautiously and regarded as exploratory until confirmed in larger multicenter studies with more balanced subgroup distributions. Likewise, although dialysis shift appeared to contribute to specific QoL domains, its association was generally weaker than that observed for educational attainment after adjustment for potential confounders.

### 5.3. Interdomain Correlations

Strong positive correlations among the WHOQOL-BREF domains reinforce the biopsychosocial model of chronic kidney disease, whereby impairments in physical health are closely associated with psychological well-being, social functioning, and environmental adaptation [16,17]. These findings highlight the importance of adopting a holistic, multidisciplinary approach to the management of patients receiving maintenance hemodialysis.

Although the correlation analysis revealed that all four WHOQOL-BREF domains were positively related to one another, these findings should be interpreted with caution. The presence of correlation does not imply that improvement in one domain automatically results in similar changes in the other domains. Patients receiving long-term hemodialysis often develop coping strategies that enable them to maintain psychological or social stability despite progressive physical decline. This adaptive response has been described in patients with chronic illnesses, in whom acceptance, resilience, family support, and effective coping mechanisms may preserve aspects of quality of life despite persistent functional limitations [16,17,18,19]. Accordingly, the correlations identified in our study should not be interpreted as evidence of direct causal relationships. Instead, they reflect the complex and multidimensional manner in which patients experience ESRD and long-term dialysis therapy. Future longitudinal studies incorporating dialysis vintage and disease duration may further clarify how these domains interact over time.

### 5.4. Impact of Dialysis Shift

Patients who received dialysis during the night shift exhibited significantly poorer QoL, particularly in the physical and environmental domains. This pattern is clinically meaningful because shift timing influences several physiological and psychosocial factors that shape patients’ overall well-being. Night shift dialysis disrupts patients’ normal circadian rhythms, often leading to chronic sleep deprivation, irregular sleep–wake cycles, and prolonged fatigue the following day. These disturbances accumulate over time, thereby reducing physical endurance, impairing daily functioning, and exacerbating symptoms such as muscle tiredness, reduced appetite, and lethargy [22,23,24,25,26].

From an environmental perspective, night shift patients also face practical challenges such as reduced availability of transportation, reliance on family members during late hours, and limited access to support services that are typically available during daytime operations. Families may struggle to accompany patients at night, thereby increasing patients’ anxiety and reducing their sense of safety and support. In addition, the availability of hospital staff and ancillary services tend to decrease during nocturnal hours, which may influence patients’ perceptions of the efficiency of care.

Psychologically, the disruption of social routines—such as missing family interactions, limited participation in evening activities, and increased isolation—may indirectly reduce patients’ motivation, coping capacity, and overall satisfaction with their treatment schedules. Patients often report feeling “out of sync” with their families and daily obligations, thus contributing to their sense of burden and reduced autonomy [22,23,26].

Although the night shift is essential to accommodate high patient volumes and maintain continuity of care in busy dialysis centers, the cumulative burden of irregular sleep, transportation difficulties, and limited social support render this shift a vulnerable time period for many patients. These findings highlight the importance of exploring flexible scheduling options, improving nighttime support services, and considering patients’ preferences in shift allocation whenever feasible. Addressing these issues may help mitigate the negative impact of night shift dialysis and ultimately enhance QoL for this subgroup.

### 5.5. International Context on Night Shift Dialysis

International evidence has also highlighted the unique challenges associated with night shift hemodialysis. Studies conducted in Saudi Arabia, Europe, Asia, and North America have consistently reported that patients who undergo dialysis overnight are more likely to report disrupted sleep patterns, reduced daytime functioning, and greater fatigue than those who received treatment on morning or afternoon schedules. In several Asian countries, where night shifts are typically used to accommodate high patient loads, researchers have linked nocturnal dialysis to poorer physical stamina and increased dependence on caregivers because of daytime exhaustion [22,23,24,25,26].

European studies evaluating nocturnal in-center and home hemodialysis have demonstrated that although extended-hour dialysis may improve selected metabolic and cardiovascular outcomes, patients continue to report sleep disturbances, social isolation, and reduced opportunities for family interaction [25,26,29]. Likewise, studies have shown that environmental barriers—including nighttime travel, transportation limitations, and reduced access to support services—contribute to lower patient satisfaction and poorer environmental quality of life scores [23,24].

Across all regions, night shift dialysis is generally adopted out of necessity rather than as a preference. Studies worldwide have consistently demonstrated that treatment at night is disadvantageous to patients in terms of their physical well-being, fatigue recovery, and overall QoL [22,23,24,25,26]. These international patterns are closely in line with our findings, thereby reinforcing the claim that the negative impact of night shift dialysis is not unique to our population but rather reflects a broader global challenge in hemodialysis scheduling.

The findings relating to night shift dialysis should also be interpreted within the context of local healthcare delivery. In many Western countries, routine maintenance hemodialysis is predominantly scheduled during daytime or early evening hours, whereas late-night sessions are comparatively uncommon. In contrast, night shift dialysis is frequently used in our institution to accommodate increasing patient numbers and optimize resource utilization. Consequently, the lower QoL observed among night shift patients may reflect organizational and contextual factors—including sleep disruption, transportation challenges, and reduced opportunities for social participation—rather than the dialysis procedure itself [22,23,24,25,26].

### 5.6. International Comparison

To place our findings within an international context, we compared our results with methodologically comparable single-center studies conducted in Poland, India, and South Africa. These studies were selected because they employed the WHOQOL-BREF instrument and similar study designs while representing diverse healthcare systems and socioeconomic settings. This approach provides a balanced international perspective and facilitates a more meaningful interpretation of quality of life outcomes among patients undergoing maintenance hemodialysis across different healthcare and socioeconomic contexts [27,29,30] (Figure 8).

Overall, our cohort demonstrated an intermediate-to-favorable quality of life profile relative to the selected international studies. Although domain-specific differences were observed, the overall pattern suggests that quality of life among patients receiving maintenance hemodialysis in our center is influenced by a combination of healthcare accessibility, socioeconomic conditions, cultural factors, and dialysis service delivery.

The physical domain showed better performance than that reported in the Indian [27] and South African [30] cohorts but remained lower than that observed in the Polish study [29]. This pattern suggests that, while physical functioning among our patients appears relatively preserved compared with other middle-income settings, opportunities remain to improve rehabilitation, exercise programs, and multidisciplinary supportive care. The superior physical outcomes reported in the Polish cohort may reflect greater availability of structured rehabilitation services and integrated multidisciplinary management, both of which have been associated with improved physical functioning among hemodialysis patients.

The psychological domain demonstrated one of the most favorable findings in our cohort compared with the selected international studies. The higher psychological quality of life observed in our patients may reflect stronger family support, cultural and religious coping strategies, and easier access to healthcare services. In contrast, poorer psychological outcomes reported in some international cohorts have been linked to greater socioeconomic hardship, higher psychological distress, limited mental health support, and a greater burden of chronic comorbidities. These findings emphasize the importance of psychosocial support as an integral component of comprehensive hemodialysis care.

Social quality of life demonstrated a more moderate pattern. Although our cohort performed more favorably than some resource-limited settings, social outcomes remained below those reported in the European cohort. This finding suggests that social well-being among hemodialysis patients is influenced not only by medical care but also by employment opportunities, social participation, caregiver dependence, and the availability of structured patient support programs. Strengthening social support services and community-based rehabilitation programs may therefore represent important opportunities for improving patient-centered outcomes.

The environmental domain showed the greatest similarity to the European cohort and substantially better outcomes than those reported in the Indian and South African studies. These findings likely reflect differences in healthcare infrastructure, transportation, accessibility of dialysis services, governmental support, and the availability of healthcare resources. Conversely, lower environmental quality of life reported in resource-constrained settings may be influenced by barriers to healthcare access, transportation difficulties, financial burden, and limitations in healthcare infrastructure. These observations highlight the important contribution of health system organization to patients’ daily experiences and treatment adherence.

Collectively, these international comparisons indicate that the quality of life of hemodialysis patients is strongly influenced by both individual and health-system factors. While our cohort demonstrated relatively favorable psychological and environmental outcomes, opportunities remain to improve physical rehabilitation and social support. These findings underscore the importance of multidisciplinary care, patient-centered rehabilitation programs, psychosocial support, and continued investment in healthcare infrastructure to optimize quality of life among patients receiving maintenance hemodialysis [27,29,30].

Beyond providing an international comparison, this study contributes contemporary evidence regarding quality of life among maintenance hemodialysis patients in the Eastern Province of Saudi Arabia, where published data remain limited. The use of the WHOQOL-BREF instrument enabled meaningful comparison with international cohorts while capturing multiple dimensions of patient well-being within the local healthcare and cultural context. Furthermore, the identification of education, age, sex, and dialysis shift as factors associated with quality of life provides clinically relevant information that may guide future interventions, service planning, and quality-improvement initiatives aimed at improving outcomes for patients receiving maintenance hemodialysis.

## 6. Strengths and Limitations

This study has several notable strengths. First, it utilized the WHOQOL-BREF, a widely validated and internationally recognized instrument for assessing quality of life, and demonstrated excellent internal consistency within the study population (Cronbach’s α = 0.946). Second, it evaluated multiple dimensions of quality of life, including physical, psychological, social, and environmental domains, providing a comprehensive assessment of patient well-being. Third, it examined the association between dialysis shift schedules and quality of life, an area that has received limited attention in the hemodialysis literature, particularly in the Middle East. Fourth, the inclusion of multivariable regression analysis allowed adjustment for potential confounding sociodemographic factors and facilitated the identification of independent predictors of quality of life outcomes. Finally, the study contributes important data from Saudi Arabia, where evidence regarding quality of life among maintenance hemodialysis patients remains relatively limited, thereby adding valuable regional information to the international literature.

This study has several limitations that should be considered when interpreting the findings. First, the study was conducted at a single tertiary-care hemodialysis center and included a relatively small sample size, which may limit the representativeness of the study population and reduce the generalizability of the findings to other hemodialysis populations, healthcare settings, and geographic regions. Although the sample was sufficient to meet the calculated study requirements, using a larger sample could have improved statistical power and enhanced the precision of subgroup analyses.

Second, the cross-sectional design captures quality of life at a single point in time and therefore does not permit the establishment of temporal or causal relationships between patient characteristics, dialysis-related factors, and quality of life outcomes. Consequently, the observed associations should be interpreted as correlational rather than causal.

Third, although multivariable regression analysis was performed to adjust for key sociodemographic factors, several clinically important variables were not consistently available for all participants and therefore could not be included in the final model. These variables include dialysis vintage, comorbidity burden, hemoglobin levels, serum albumin levels, vascular access type, sleep quality, and intradialytic symptoms. Previous studies have shown that these factors may substantially influence quality of life outcomes among patients receiving maintenance hemodialysis. Consequently, the omission of these variables may have resulted in residual confounding and could have influenced the observed associations between dialysis-shift schedules, patient characteristics, and quality of life domains.

Some subgroup analyses within the multinomial logistic regression models were based on small numbers of participants within certain categories, which may have resulted in unstable estimates and limited the reliability of these comparisons. Therefore, these regression findings should be considered exploratory and interpreted with caution until validated in large studies with more balanced subgroup distributions.

Finally, although all eligible patients were consecutively recruited, 17 patients were excluded because of predefined exclusion criteria or refusal to participate. Therefore, selection bias cannot be completely excluded. The characteristics of excluded participants may have differed from those included in the final analysis, potentially influencing the observed quality of life outcomes. Therefore, the findings should be interpreted with appropriate caution and may not be fully generalizable to all patients receiving maintenance hemodialysis.

## 7. Clinical and Public Health Implications

Routine QoL assessments should be incorporated into dialysis care, as recommended in international guidelines. Tailored interventions are needed for vulnerable subgroups, including women and patients with low levels of education. Rehabilitation programs, psychosocial counseling, and flexible shift scheduling may meaningfully improve QoL. Policy-makers should consider efforts to strengthen community-based support services with the aim of reducing social isolation.

## 8. Recommendations

### 8.1. Incorporate Structured Physical Activity into Routine Dialysis Care

Physical health was the most strongly affected domain in this study, which mirrors the findings reported by studies conducted in other regions. Exercise is a safe and effective intervention for patients receiving hemodialysis. Relevant programs can include intradialytic cycling, light resistance exercises, balance training, and individualized progression depending on the patients’ ability. Regular activity promotes better stamina, reduces fatigue, improves muscle strength, and contributes to psychological well-being and increased independence.

### 8.2. Review and Optimize Dialysis Shift Allocation

Night shift dialysis was linked to lower QoL scores, particularly in the physical and environmental domains. Whenever possible, vulnerable patients should be scheduled to undergo treatment during daytime shifts to support better sleep patterns, family routines, and work commitments. Greater attention to patient preference and the expansion of daytime capacity can help minimize the burden associated with night sessions. The center has already begun to address this issue by gradually reducing the availability of night shift treatment.

### 8.3. Strengthen Patient Education and Health Literacy

Education plays a central role in improving patients’ ability to manage their illness. Structured educational sessions concerning ESRD, diet, symptom monitoring, medication adherence, and the benefits of physical activity can help enhance patients’ coping ability, reduce their anxiety, and improve their QoL scores. Clear communication and patient-tailored teaching materials are essential in this context.

### 8.4. Implement Routine Quality of Life Assessments

Regular QoL assessments allow clinicians to detect early decline and intervene before symptoms worsen. The use of validated tools such as the WHOQOL-BREF every 6–12 months can help identify changes in physical comfort, emotional health, social engagement, and environmental needs. The integration of QoL monitoring into routine care can help encourage more patient-centered decision-making and support targeted interventions.

### 8.5. Enhance Psychosocial and Social Support Services

Psychosocial support should be viewed as a core component of dialysis care. Counseling, peer-support groups, caregiver education, and social-service referrals can help patients manage stress, isolation, and emotional fatigue. These services are particularly important for vulnerable groups, including women and individuals who receive only limited social support.

### 8.6. Future Research

Further studies are needed to explore the long-term effects of exercise programs, shift scheduling, and educational interventions on QoL. Larger multicenter studies and qualitative interviews could help capture the full range of challenges faced by hemodialysis patients and inform national renal care strategies.

## 9. Conclusions

The principal contribution of this study is the identification of independent sociodemographic determinants of domain-specific quality of life among patients receiving maintenance hemodialysis. Multivariable regression analysis demonstrated that educational attainment, age, sex, and dialysis shift were independently associated with specific WHOQOL-BREF domains, highlighting that quality of life is influenced by factors extending beyond the clinical burden of end-stage kidney disease. These findings support the routine incorporation of multidomain quality of life assessment into hemodialysis practice to facilitate the early identification of vulnerable patients and guide individualized interventions targeting physical functioning, psychological well-being, patient education, and dialysis service delivery. Although the cross-sectional design precludes causal inference, the results provide a foundation for future multicenter longitudinal studies to validate these associations and evaluate interventions aimed at improving patient-reported outcomes in maintenance hemodialysis populations.

## Figures and Tables

**Table 1:** Demographic characteristics of the participants.

Demographics	Frequency	Percentage
**Sex**		
Female	46	58.2
Male	33	41.8
**Age**		
20–30	2	2.5
30–50	16	20.3
50–70	41	51.9
Older than 70	20	25.3
**Level of Education**		
Illiterate	35	44.3
Middle school	4	5.1
Primary school	17	21.5
Secondary school	14	17.7
University	9	11.4
**Employment Status**		
Employed	4	5.1
Retired	26	32.9
Unemployed	49	62
**Shift**		
Afternoon	18	22.8
Evening	18	22.8
Morning	25	31.6
Night	18	22.8
**Marital Status**		
Divorced	4	5.1
Married	50	63.3
Single	5	6.3
Widowed	20	25.3

**Table 2:** Distribution of participants’ quality of life (QOL) ratings across the four WHOQOL-BREF domains.

QOL	Very Poor	Poor	Moderate	Good	Very Good
**Physical**	-	32 (40.5%)	24 (30.4%)	21 (26.6%)	2 (2.5%)
**Psychological**	-	8 (10.1%)	33 (41.8%)	24 (30.4%)	14 (17.7%)
**Social**	18 (22.8%)	19 (24.1%)	18 (22.8%)	18 (22.8%)	6 (7.5%)
**Environment**	1 (1.3%)	6 (7.6%)	22 (27.9%)	26 (32.9%)	24 (30.3%)

**Table 3:** Domain means and transformed scores.

QOL	Mean ± SD	Transformed Mean ± SD
**Physical**	2.48 ± 0.93	49.69 ±18.77
**Psychological**	3.19 ± 0.95	63.83 ± 19.15
**Social**	2.40 ± 1.15	48.02 ± 24.92
**Environment**	3.37 ± 0.96	67.56 ± 19.27

**Table 4:** The correlations among the WHOQOL-BREF domains.

Spearman’s Correlation						
			Physical	Psychological	Social	Environment
**Spearman’s rho**	**Physical**	Coefficient	1.000	0.729 **	0.639 **	0.718 **
		Sig. (2-tailed)		0.000	0.000	0.000
	**Psychological**	Coefficient	0.729 **	1.000	0.723 **	0.774 **
		Sig. (2-tailed)	0.000		0.000	0.000
	**Social**	Coefficient	0.639 **	0.723 **	1.000	0.715 **
		Sig. (2-tailed)	0.000	0.000		0.000
	**Environment**	Coefficient	0.718 **	0.774 **	0.715 **	1.000
		Sig. (2-tailed)	0.000	0.000	0.000	

** Correlation is significant at the 0.01 level (2-tailed).

**Table 5:** The associations between sociodemographic characteristics and the social QoL domain (WHOQOL-BREF).

Variable	Category	Mean ± SD	*n* (%)	*p* Value
Sex	Female	37.50 ± 43.83	4 (5.3%)	
Age group	50–70	25.00 ± 11.79	2 (2.7%)	

**Table 6:** The associations between sociodemographic characteristics and the psychological QoL domain (WHOQOL-BREF).

Variable	Category	Mean ± SD	*n* (%)	*p* Value
Sex	Female	59.38 ± 23.91	4 (5.5%)	
Age group	50–70	37.50 ± nan	1 (1.4%)	

**Table 7:** The associations between sociodemographic characteristics and the physical QoL domain (WHOQOL-BREF).

Variable	Category	Mean ± SD	*n* (%)	*p* Value
Sex	Female	32.76 ± 22.25	46 (63.0%)	0.005
Sex	Male	48.54 ± 23.07	27 (37.0%)	0.005
Age Group	50–70	36.84 ± 23.95	38 (52.1%)	0.105
Age Group	30–50	45.98 ± 22.99	16 (21.9%)	0.105
Age Group	Older than 70	32.14 ± 21.47	17 (23.3%)	0.105
Age Group	20–30	67.86 ± 20.20	2 (2.7%)	0.105
Level of Education	Primary school	43.08 ± 20.56	16 (21.9%)	0.001
Level of Education	Illiterate	25.71 ± 20.60	30 (41.1%)	0.001
Level of Education	Secondary school	54.85 ± 18.83	14 (19.2%)	0.001
Level of Education	Middle school	36.61 ± 23.94	4 (5.5%)	0.001
Level of Education	University	49.21 ± 25.86	9 (12.3%)	0.001
Employment Status	Unemployed	33.52 ± 23.34	44 (60.3%)	0.101
Employment Status	Retired	45.19 ± 21.82	23 (31.5%)	0.101
Employment Status	Employed	49.11 ± 30.50	4 (5.5%)	0.101
Marital Status	Married	41.59 ± 22.54	45 (61.6%)	0.085
Marital Status	Single	53.57 ± 24.61	5 (6.8%)	0.085
Marital Status	Widowed	28.01 ± 22.97	19 (26.0%)	0.085
Marital Status	Divorced	36.61 ± 28.33	4 (5.5%)	0.085

**Table 8:** The associations between sociodemographic characteristics and the environment QoL domain (WHOQOL-BREF).

Variable	Category	Mean ± SD	*n* (%)	*p* Value
Sex	Female	68.75 ± 17.49	4 (5.6%)	
Age group	50–70	53.12 ± nan	1 (1.4%)	

**Table 9:** The multinomial logistic regression for the physical domain.

Physical Domain is Set as the Dependent Variable with the Reference Group of “Very Good”.		*p* Value	Odd Ratio	95% Confidence Interval for Odd Ratio	
				Lower Bound	Upper Bound
**Poor vs. Very Good**	[Level of Education = Illiterate vs. University]	1.000	8.333	0.000	b
	[Level of Education = Middle school vs. University]	1.000	0.635	0.000	b
	[Level of Education = Primary school vs. University]	0.001	4.447 × 10^−8^	7.799 × 10^−10^	2.536 × 10^−6^
	[Level of Education = Secondary school vs. University]	0.001	5.303 × 10^−9^	2.332 × 10^−10^	1.206 × 10^−7^
	[Shift = Afternoon vs. Night]	0.996	5.818 × 10^−9^	0.000	b
	[Shift = evening vs. Night]	0.996	1.057 × 10^−7^	0.000	b
	[Shift = Morning vs. Night]	0.166	0.177	0.015	2.049
**Moderator vs. Very Good**	[Level of Education = Illiterate vs. University]	1.000	0.402	0.000	b
	[Level of Education = Middle school vs. University]	1.000	0.912	0.000	b
	[Level of Education = Primary school vs. University]	0.001	2.391 × 10^−8^	4.858 × 10^−10^	1.177 × 10^−6^
	[Level of Education = Secondary school vs. University]	0.001	1.060 × 10^−8^	1.099 × 10^−9^	1.023 × 10^−7^
	[Shift = Afternoon vs. Night]	0.996	2.707 × 10^−8^	0.000	b
	[Shift = evening vs. Night]	0.996	3.232 × 10^−8^	0.000	b
	[Shift = Morning vs. Night]	0.464	2.119	0.283	15.851
**Good vs. Very Good**	[Level of Education = Illiterate vs. University]	0.999	0.039	0.000	b
	[Level of Education = Middle school vs. University]	1.000	0.148	0.000	b
	[Level of Education = Primary school vs. University]	0.001	2.072 × 10^−9^	5.934 × 10^−11^	7.232 × 10^−8^
	[Level of Education = Secondary school vs. University]		5.856 × 10^−9^	5.856 × 10^−9^	5.856 × 10^−9^
	[Shift = Afternoon vs. Night]	0.996	7.405 × 10^−8^	0.000	b
	[Shift = Evening vs. Night]	0.996	1.329 × 10^−8^	0.000	b
	[Shift = Morning vs. Night]		3.771	3.771	3.771

The fit of the final model exhibited a significant improvement over that of the intercept only, including a pseudo-R square of 0.569, thus indicating a moderately strong model. Note: b = Parameter estimates could not be computed because of very small sample sizes or zero observations in one or more outcome categories, resulting in complete or quasi-complete separation.

**Table 10:** The multinomial logistic regression for the psychological domain.

Psychological Domain is Set as the Dependent Variable with the Reference Group of “Very Good”.		*p* Value	Odd Ratio	95% Confidence Interval for Odd Ratio	
				Lower Bound	Upper Bound
**Poor vs. Very Good**	[Age Group = 20–30 vs. Older than 70]		6.318	6.318	6.318
	[Age Group = 30–50 vs. Older than 70]	0.997	6.464 × 10^−7^	0.000	b
	[Age Group = 50–70 vs. Older than 70]	0.860	1.223	0.130	11.489
	[Level of Education = Illiterate vs. University]	0.997	172,513,795.628	0.000	b
	[Level of Education = Middle school vs. University]	0.999	53,228,682.024	0.000	b
	[Level of Education = Primary school vs. University]	0.998	42,101,338.758	0.000	b
	[Level of Education = Secondary school vs. University]	1.000	1.033	0.000	b
**Moderate vs. Very Good**	[Age Group = 20–30 vs. Older than 70]	0.998	63,003,642.543	0.000	b
	[Age Group = 30–50 vs. Older than 70]	0.536	1.969	0.230	16.822
	[Age Group = 50–70 vs. Older than 70]	0.664	0.692	0.132	3.632
	[Level of Education = Illiterate vs. University]	0.011	21.083	1.984	224.022
	[Level of Education = Middle school vs. University]	0.998	192,977,815.803	0.000	b
	[Level of Education = Primary school vs. University]	0.107	7.717	0.645	92.347
	[Level of Education = Secondary school vs. University]	0.648	1.688	0.179	15.913
**Good vs. Very Good**	[Age Group = 20–30 vs. Older than 70]	0.998	78,587,458.601	0.000	b
	[Age Group = 30–50 vs. Older than 70]	0.193	4.171	0.486	35.787
	[Age Group = 50–70 vs. Older than 70]	0.839	1.203	0.201	7.201
	[Level of Education = Illiterate vs. University]				
	[Level of Education = Middle school vs. University]	0.157	5.455	0.519	57.332
	[Level of Education = Primary school vs. University]	0.998	120,998,869.109	0.000	b
	[Level of Education = Secondary school vs. University]	0.086	7.863	0.748	82.696
	[Age Group = 20–30 vs. Older than 70]	0.684	1.540	0.192	12.337

The fit of the final model exhibited a significant improvement over that of the intercept only, including a pseudo-R square of 0.334, thus indicating a moderately strong model. Note: b = Parameter estimates could not be computed because of very small sample sizes or zero observations in one or more outcome categories, resulting in complete or quasi-complete separation.

**Table 11:** The multinomial logistic regression for the social domain.

Social Domain is Set as the Dependent Variable with the Reference Group of “Very Good”.		*p* Value	Odd Ratio	95% Confidence Interval for Odd Ratio	
				Lower Bound	Upper Bound
**Very Poor vs. Very Good**	[Sex = Female vs. Male]	0.019	38.718	1.835	816.981
	[Employment Status = Employed vs. Unemployed]		14.031	14.031	14.031
	[Employment Status = Retired vs. Unemployed]	0.867	1.243	0.097	15.950
**Poor vs. Very Good**	[Sex = Female vs. Male]	0.727	1.510	0.150	15.179
	[Employment Status = Employed vs. Unemployed]		1.031	1.031	1.031
	[Employment Status = Retired vs. Unemployed]	0.784	0.732	0.079	6.783
**Moderate vs. Very Good**	[Sex = Female vs. Male]	0.590	1.901	0.184	19.651
	[Employment Status = Employed vs. Unemployed]		1.256	1.256	1.256
	[Employment Status = Retired vs. Unemployed]	0.939	0.916	0.096	8.728
**Good vs. Very Good**	[Sex = Female vs. Male]	0.176	5.688	0.458	70.615
	[Employment Status = Employed vs. Unemployed]	0.998	3,251,248,222.978	0.000	b
	[Employment Status = Retired vs. Unemployed]	0.547	2.112	0.186	24.022

The fit of the final model exhibited a significant improvement over that of the intercept only, including a pseudo-R square of 0.324, thus indicating a moderately strong model. Note: b = Parameter estimates could not be computed because of very small sample sizes or zero observations in one or more outcome categories, resulting in complete or quasi-complete separation.

**Table 12:** The multinomial logistic regression for the environment domain.

Environment Domain is Set as the Dependent Variable with the Reference Group of “Very Good”.		*p* Value	Odd Ratio	95% Confidence Interval for Odd Ratio	
				Lower Bound	Upper Bound
**Very Poor vs. Very Good**	[Age Group = 20–30 vs. Older than 70]		242,534.857	242,534.857	242,534.857
	[Age Group = 30–50 vs. Older than 70]	0.998	877,832.724	0.000	b
	[Age Group = 50–70 vs. Older than 70]	0.995	1,167,868.707	0.000	b
	[Level of Education = Illiterate vs. University]				
	[Level of Education = Middle school vs. University]	0.998	13,215,955.017	0.000	b
	[Level of Education = Primary school vs. University]		12,221,530,058,038.713	12,221,530,058,038.713	12,221,530,058,038.713
	[Level of Education = Secondary school vs. University]	1.000	3.037	0.000	b
	[Shift = Afternoon vs. Night]	0.998	10,118,958.042	0.000	b
	[Shift = evening vs. Night]	1.000	8.640	0.000	b
	[Shift = Morning vs. Night]	1.000	0.867	0.000	b
**Poor vs. Very Good**	[Age Group = 20–30 vs. Older than 70]	0.998	7.057 × 10^−8^	0.000	b
	[Age Group = 30–50 vs. Older than 70]	0.995	1.201 × 10^−7^	0.000	b
	[Age Group = 50–70 vs. Older than 70]	0.167	0.191	0.018	1.997
	[Level of Education = Illiterate vs. University]				
	[Level of Education = Middle school vs. University]	0.995	57,407,561.768	0.000	b
	[Level of Education = Primary school vs. University]	0.997	105,522,337,118,942.060	0.000	b
	[Level of Education = Secondary school vs. University]	0.995	49,556,924.883	0.000	b
	[Shift = Afternoon vs. Night]	0.063	0.071	0.004	1.157
	[Shift = evening vs. Night]	0.928	0.862	0.034	21.727
	[Shift = Morning vs. Night]	0.993	2.102 × 10^−8^	0.000	b
**Moderate vs. Very Good**	[Age Group = 20–30 vs. Older than 70]	0.997	1.717 × 10^−7^	0.000	b
	[Age Group = 30–50 vs. Older than 70]	0.675	0.483	0.016	14.525
	[Age Group = 50–70 vs. Older than 70]	0.890	0.877	0.135	5.679
	[Level of Education = Illiterate vs. University]	0.993	115,953,032.064	0.000	b
	[Level of Education = Middle school vs. University]	0.993	767,930,637,412,775.200	0.000	b
	[Level of Education = Primary school vs. University]	0.993	59,794,058.372	0.000	b
	[Level of Education = Secondary school vs. University]	0.994	5,167,927.544	0.000	b
	[Shift = Afternoon vs. Night]	0.368	0.362	0.040	3.305
	[Shift = evening vs. Night]	0.149	7.075	0.498	100.577
	[Shift = Morning vs. Night]	0.961	1.055	0.126	8.819
**Good vs. Very Good**	[Age Group = 20–30 vs. Older than 70]	0.889	0.789	0.028	22.209
	[Age Group = 30–50 vs. Older than 70]	0.806	0.772	0.098	6.055
	[Age Group = 50–70 vs. Older than 70]	0.022	0.128	0.022	0.744
	[Level of Education = Illiterate vs. University]	0.270	4.418	0.316	61.801
	[Level of Education = Middle school vs. University]	0.995	75,391,712.880	0.000	b
	[Level of Education = Primary school vs. University]	0.081	9.851	0.755	128.540
	[Level of Education = Secondary school vs. University]	0.441	2.395	0.260	22.034
	[Shift = Afternoon vs. Night]	0.022	0.062	0.006	0.671
	[Shift = evening vs. Night]	0.776	1.375	0.153	12.374
	[Shift = Morning vs. Night]	0.197	0.288	0.044	1.911

The fit of the final model exhibited a significant improvement over that of the intercept only, including a pseudo-R square of 0.618, thus indicating a moderately strong model. Note: b = Parameter estimates could not be computed because of very small sample sizes or zero observations in one or more outcome categories, resulting in complete or quasi-complete separation.

## Data Availability

The data presented in this study are available upon request from the corresponding author due to ethical reasons. The dataset contains information collected from hemodialysis patients and cannot be made publicly available because of participant confidentiality and institutional ethical requirements.

## Abbreviations

The following abbreviations are used in this manuscript:

ESRD	End-Stage Renal Disease
QoL	Quality of Life
WHOQOL-BREF	World Health Organization Quality of Life-BREF
CKD	Chronic Kidney Disease
MNGHA	Ministry of National Guard Health Affairs
KAIMRC	King Abdullah International Medical Research Centre
IRB	Institutional Review Board
ANOVA	Analysis of Variance
SD	Standard Deviation
OR	Odds Ratio
CI	Confidence Interval

## References

[1] (2024). Kidney Disease: Improving Global Outcomes CKD Work Group. KDIGO 2024 clinical practice guideline for the evaluation and management of chronic kidney disease. Kidney Int..

[2] GBD Chronic Kidney Disease Collaboration (2020). Global, regional, and national burden of chronic kidney disease, 1990–2017: A systematic analysis for the Global Burden of Disease Study 2017. Lancet.

[3] Hill N.R., Fatoba S.T., Oke J.L., Hirst J.A., O’Callaghan C.A., Lasserson D.S., Hobbs F.R. (2016). Global prevalence of chronic kidney disease: A systematic review and meta-analysis. PLoS ONE.

[4] Kim S.G., Lee I.H. (2023). The impact of quality of life measured by WHOQOL-BREF on mortality in maintenance hemodialysis patients: A single-center retrospective cross-sectional study. J. Yeungnam Med. Sci..

[5] Ranabhat K., Khanal P., Mishra S.R., Khanal A., Tripathi S., Sigdel M.R. (2020). Health-related quality of life among haemodialysis and kidney transplant recipients from Nepal: A cross-sectional study using WHOQOL-BREF. BMC Nephrol..

[6] Joshi U., Subedi R., Poudel P., Ghimire P.R., Panta S., Sigdel M.R. (2017). Assessment of quality of life in patients undergoing hemodialysis using WHOQOL-BREF questionnaire: A multicenter study. Int. J. Nephrol. Renov. Dis..

[7] The WHOQOL Group (1995). The World Health Organization quality of life assessment: Position paper from the World Health Organization. Soc. Sci. Med..

[8] The WHOQOL Group (1998). Development of the World Health Organization WHOQOL-BREF quality of life assessment. Psychol. Med..

[9] World Health Organization (1996). WHOQOL-BREF: Introduction, Administration, Scoring and Generic Version of the Assessment.

[10] Liu X., Meng J., Zhang S. (2025). Analysis of factors influencing quality of life in hemodialysis patients based on KDQOL-36: A cross-sectional study. Int. J. Artif. Organs.

[11] Lalo R., Kamberi F., Stasa E., Lalo K. (2025). Burden of hemodialysis on health-related quality of life: Insights from a multi-center cross-sectional analysis in Southern Albania. Front. Med..

[12] Sułkowski L., Matyja A., Matyja M. (2024). Social support and quality of life in hemodialysis patients: A Comparative Study with Healthy Controls. Medicina.

[13] Alqalah T.A.H., Alrubaiee G.G., Alkubati S.A. (2025). Factors associated with the quality of life and needs of hemodialysis patients in Saudi Arabia: A basis for improved care. Medicina.

[14] Alshehri M., Alshehri A., Alfageeh A., Asiri K., Alshehri A., Alqahtani F., Alshehri M., Alshabab M., Asiri O. (2023). Who have a better kidney-related quality of life: Peritoneal dialysis or hemodialysis patients? A cross-sectional study from Saudi Arabia. BMC Nephrol..

[15] Asiri W.M.A., Almutlaq A.H., Almutairi K.H., Alshahrani M.S.M., Asiri A.A.H., Alshahrani R.S., Soliman O.H.M. (2022). Quality of life and mental health among hemodialysis patients in Aseer Region, Saudi Arabia. Bahrain Med. Bull..

[16] van Oevelen M., Bonenkamp A.A., van Eck van der Sluijs A., Bos W.J.W., Douma C.E., van Buren M., Meuleman Y., Dekker F.W., van Jaarsveld B.C., Abrahams A.C. (2024). Health-related quality of life and symptom burden in patients on haemodialysis. Nephrol. Dial. Transplant..

[17] Raoofi S., Pashazadeh Kan F., Rafiei S., Hoseinipalangi Z., Rezaei S., Ahmadi S., Sharifi A., Shabaninejad H., Benis M.R., Ghashghaee A. (2023). Global health-related quality of life in patients undergoing dialysis: A systematic review and meta-analysis. BMJ Support Palliat. Care.

[18] Al-Mutary H., Bonner A., Douglas C. (2013). Symptom burden and quality of life in chronic kidney disease: A review of recent literature. J. Ren. Care.

[19] Alshogran O.Y., Shatnawi E.A., Atawalbeh S.M., Jarab A.S., Farah R.I. (2021). Predictors of poor health-related quality of life among hemodialysis patients with anemia in Jordan. Health Qual. Life Outcomes.

[20] Abbas E.M., Harshavardhan R., Mohammed H., Loona V., Faseeh K.M. (2024). An assessment of quality of life in ESRD patients undergoing hemodialysis. Egypt. J. Intern. Med..

[21] Hays R.D., Kallich J.D., Mapes D.L., Coons S.J., Carter W.B. (1994). Development of the Kidney Disease Quality of Life instrument. Qual. Life Res..

[22] Nowrooz S., Alanazi T., Al-Ghamdi A., Alzahrani A., Alshammari A., AlYaqoot N., Almutraid M., Jaradat A., El-Agroudy A. (2023). Quality of life among hemodialysis patients: Role of the dialysis shift. Saudi J. Kidney Dis. Transpl..

[23] Hariharan N., Chkhikvadze L., Mateshvili A., Sebastian A.M., Mathew N.S., Shavgulidze E., Tchokhonelidze I. (2024). Association of sleep problems with dialysis shifts in patients undergoing hemodialysis in Tbilisi, Georgia. Cureus.

[24] Elsayed M.M., Zeid M.M., Hamza O.M.R., Elkholy N.M. (2022). Dialysis recovery time: Associated factors and its association with quality of life of hemodialysis patients. BMC Nephrol..

[25] Dumaine C.S., Ravani P., Parmar M.K., Leung K.C.W., MacRae J.M. (2022). In-center nocturnal hemodialysis improves health-related quality of life for patients with end-stage renal disease. J. Nephrol..

[26] Hull K.L., Quann N., Glover S., Wimbury C., Churchward D.R., Pickering W.P., Preston R., Baines R., Graham-Brown M.P., Burton J.O. (2021). Evaluating the clinical experience of a regional in-center nocturnal hemodialysis program: The patient and staff perspective. Hemodial. Int..

[27] Sathvik B.S., Parthasarathi G., Narahari M.G., Gurudev K.C. (2008). An assessment of the quality of life in hemodialysis patients using the WHOQOL-BREF questionnaire. Indian J. Nephrol..

[28] Ramasamy S., Rathore V., Agrawal V., Galhotra A. (2025). Burden level and quality of life of caregivers of hemodialysis patients in central India: A mixed-method cross-sectional study. BMC Res. Notes.

[29] Dembowska E., Jaroń A., Gabrysz-Trybek E., Bladowska J., Gacek S., Trybek G. (2022). Quality of life in patients with end-stage renal disease undergoing hemodialysis. J. Clin. Med..

[30] Mbeje P.N. (2022). Factors affecting the quality of life for patients with end-stage renal disease on dialysis in KwaZulu-Natal province, South Africa: A descriptive survey. Health SA Gesondheid.

